# Case report: From sequence to solution: tailoring treatment for transformed follicular lymphoma (DLBCL) through next generation sequencing study

**DOI:** 10.3389/fonc.2024.1308492

**Published:** 2024-02-29

**Authors:** Antonin Bouroumeau, Sarah Perdikis-Prati, Noémie Lang

**Affiliations:** ^1^ Division of Clinical Pathology, Diagnostic Department, Geneva University Hospital, Geneva, Switzerland; ^2^ Department of Oncology, Geneva University Hospital, Geneva, Switzerland; ^3^ Center of Translational Research in Oncohematology, Faculty of Medicine, University of Geneva, Geneva, Switzerland

**Keywords:** follicular lymphoma, aggressive transformation, NGS - next generation sequencing, PD-1 inhibitor, tumor mutational burden (TMB)

## Abstract

Immune checkpoint blockade (ICB) has indeed transformed the outlook for many advanced-stage solid tumors, yet its effectiveness in hematological malignancies has been particularly limited, with success predominantly demonstrated in classical Hodgkin lymphoma (cHL) and immune-privilege subtypes of non-Hodgkin lymphoma (NHL). In this report, we present an impactful case of a 71-year-old man grappling with refractory follicular lymphoma (rFL) that had progressed to a high-grade lymphoma, leaving no conventional treatment options on the table. Notably, the histological examination of the tumor tissue revealed a markedly elevated PD-L1 expression, illuminating the potential for immunotherapy to be effective. Additionally, comprehensive gene sequencing unveiled a moderate tumor mutational burden (TMB), deepening our understanding of the tumor’s molecular intricacies. As his health declined with no access to cell therapies or clinical trials at that time, a combination treatment of PD-1 ICB and an anti-CD20 drug surprisingly led to a significant improvement in his condition and long-term remission. While PD-1 ICB therapy has historically shown limited responses in non-Hodgkin lymphomas (NHLs), this case serves as a beacon of optimism, underscoring the promise of combining immunotherapy modalities and the potential of comprehensive molecular assessments in charting innovative treatments for extensively treated NHL patients. The quest for predictive biomarkers to gauge treatment response remains a formidable challenge. This report serves as a testament to the ever-evolving landscape of cancer treatment, where precision medicine and immunotherapy continue to unlock new possibilities for those confronting the most challenging malignancies.

## Introduction

Follicular lymphoma (FL) constitutes a significant proportion of non-Hodgkin lymphoma (NHL) cases, primarily affecting older individuals. Despite the availability of diverse treatment modalities, FL is often considered incurable, with relapses posing a significant challenge, especially in frail patients. While immune checkpoint blockade (ICB) has revolutionized solid tumor treatment, its effectiveness in NHL, including FL, remains uncertain due to the lack of reliable predictive biomarkers.

## Case description

**Figure 1:**
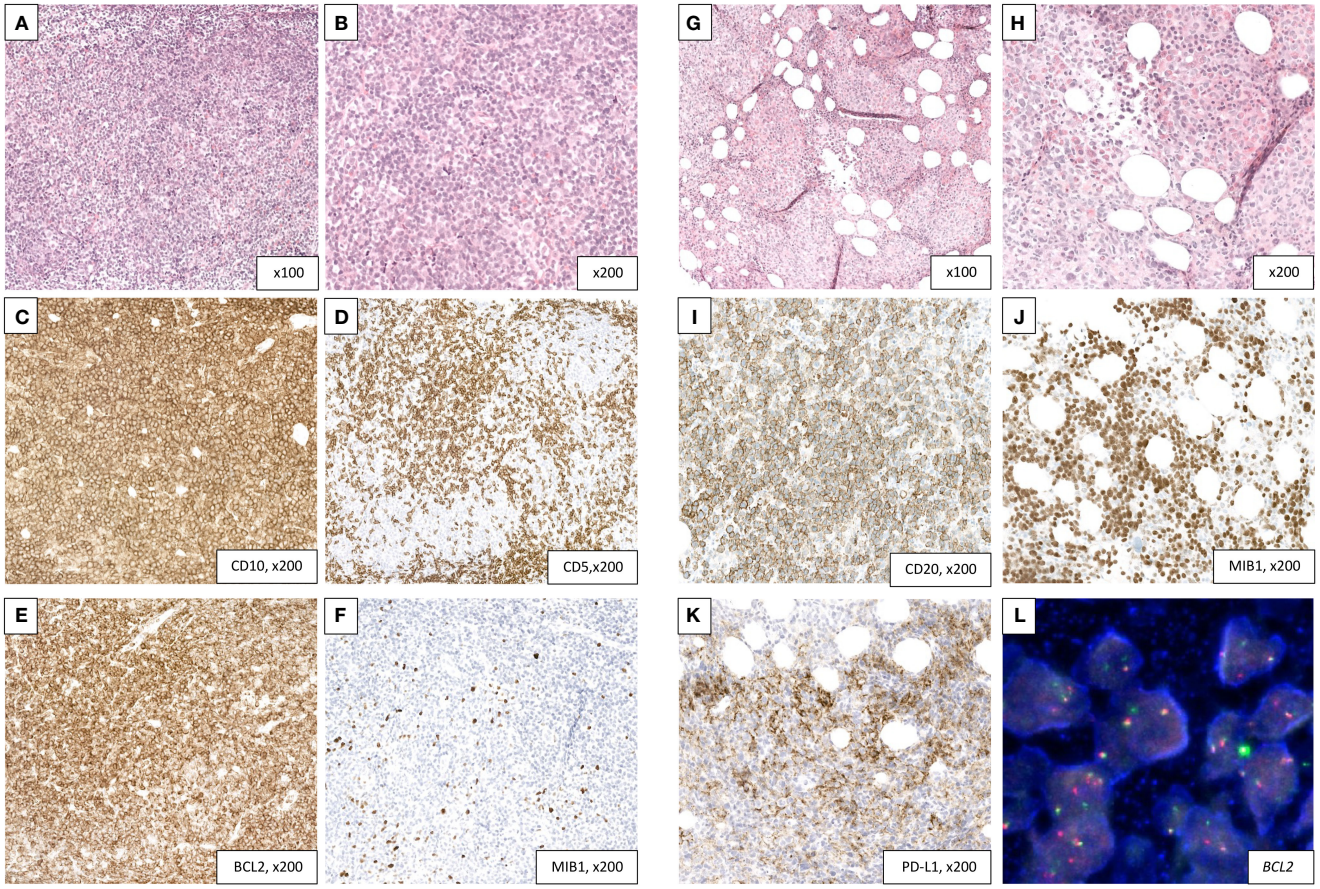
Transformation of a follicular lymphoma **(A–F)** into a DLBCL with high PD-L1 expression **(G–L)**. **(A–F**) Follicular lymphoma diagnosed on a retroperitoneal lymph node biopsy. H&E sections **(A, B)** showing a vaguely nodular lymphoid proliferation composed mainly of centrocytes with rare centroblasts (grade I). The nodular architecture is better highlighted in immunohistochemistry with a T-cell marker (CD5, **D**). The tumor cells are diffusely reactive to CD10 **(C)** and Bcl2 **(E)** with a very low proliferation index (5%, **F**). **(G–L)** Transformation into a DLBCL with PD-L1 expression in a bone marrow core biopsy. H&E sections **(G, H)** showing a replacement of the hematopoietic lineages by a diffuse lymphoid proliferation consisting of large-size and atypical cells. The tumor cells retain a CD20 positivity **(I)** with half of them showing a PD-L1 expression **(K)**. Proliferation index is high (70%, **J**). FISH analysis confirms a BCL2 rearrangement **(L)**. BCL-2, B-cell lymphoma 2; DLBCL, diffuse large B-cell lymphoma; FISH, fluorescence *in situ* hybridization; H&E, hematoxylin and eosin; PD-L1, programmed death-ligand 1.

**Figure 2:**
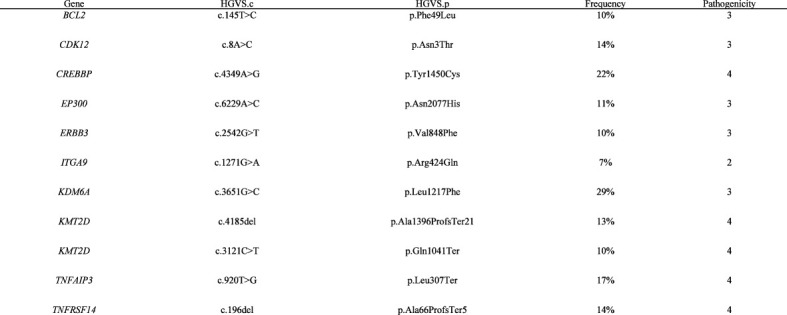
List of somatic mutations retained with a 400-gene NGS panel in the bone marrow sample. Bone marrow sample of patient was sequenced in 2020. The panel used for the sequencing was the NGS400v2, the custom capture panel based on Agilent SureSelect XT HS technology and covering all exons of 420 genes and the TERT promoter region. Sequencing was done on an Illumina NextSeq 500 System. The data were analyzed using HUG internal software (Soap 2.1.1)—Oncobench v4. with hg19 as a reference. The variant calling software detected 26 somatic mutations. The germline variants were detected by sequencing of blood or non-tumor tissue samples. Of the 26 somatic variants detected, only 16 had a potential impact on the protein, and of these 16 variants, two had an allelic frequency below 5% and were filtered out. Pathogenicity was assessed for the remaining 14 variants presented. The tumor mutation burden was 6.75 mut/Mb. Reference—ENSEMBL transcript ID, pathogenicity is classified as follows: 1, benign/2, probably benign/3, uncertain/4, probably pathogenic/5, pathogenic. HGVS, Human Genome Variation Society; HGVS.c, DNA variant, HGVS.p, protein variant; HUG, Hôpitaux Universitaires de Genève; VAF, variant allele frequency.

A retired 71-year-old man, previously diagnosed with chronic kidney disease (CKD), received an incidental diagnosis of grade 1 stage IVA follicular lymphoma (FL) an astounding 16 years ago. At the time of this diagnosis, his FLIPI score was 3/5, meeting criteria based on age, stage, and involvement of more than four nodal areas (refer to **Figures 1**, **2** for the initial radiological and pathological assessments). Notably, the GELF criteria were not initially met (GELF score, 0/7), prompting a period of close observation without any treatment, which extended for nearly 3 years.

Subsequently, his medical journey took a more intense trajectory as he underwent a series of treatments in response to the disease’s progression. These treatments included R-CHOP (rituximab, cyclophosphamide, hydroxyadriamycin, vincristine, and prednisone) with rituximab maintenance, R-ICE (rituximab, ifosfamide, carboplatin, and etoposide), rituximab-bendamustine, pelvic VMAT radiotherapy, and idelalisib. Over this extended period, the patient encountered multiple infectious complications and ultimately reached end-stage CKD, necessitating hemodialysis.

While on idelalisib, the patient’s health took a dramatic downturn, resulting in severe illness characterized by febrile grade IV pancytopenia and significant dyspnea. A PET-CT scan revealed intensely diffuse osteo-medullary hypermetabolic activity, with a mixed response in previously identified areas of lymphadenopathy. A subsequent bone marrow biopsy confirmed the diagnosis of high-grade transformation from the prior FL to diffuse large B-cell lymphoma. This transformation was characterized by a diffuse proliferation of large-sized lymphoid cells, with CD20 positivity.

Despite a poor performance status (ECOG 4) and significant comorbidities (Cumulative Illness Rating Scale (CIRS) score of 14), the patient exhibited strong determination to explore a new palliative therapeutic option. Remarkably, the histological examination of his transformed FL revealed a pronounced and widespread expression of PD-L1 by the tumor cells (TPS > 50) (PD-L1 (E1L3N) XP Rabbit mAb, Cell Signaling) (**Figure 1**). Complementary molecular analysis using a 400-gene next-generation sequencing (NGS) panel (**Supplementary Figure S1**) unveiled classical mutations commonly associated with transformed FL, including CREBBP, KMT2D, BCL2, EP300, and TNFSRF14 genes; however, these mutations were not actionable. Notably, large deletions or insertions, particularly those described in genes such as KIT, CTNNB1, TP53, CDKN2A, PTEN, and FLT3, were not detected. Furthermore, the analysis revealed a moderate tumor mutational burden (TMB) of 6.75 mutations per megabase (mut/Mb) (**Figure 2**). Furthermore, we conducted Oncoscan Affymetrix analysis, which unveiled gains in 17q, 18q, and 18p, along with loss of heterozygosity (LOH) in Xq and Xp. Interestingly, none of these identified anomalies exhibited a direct correlation with the expression of PD1/PD-L1.

**Figure 3:**
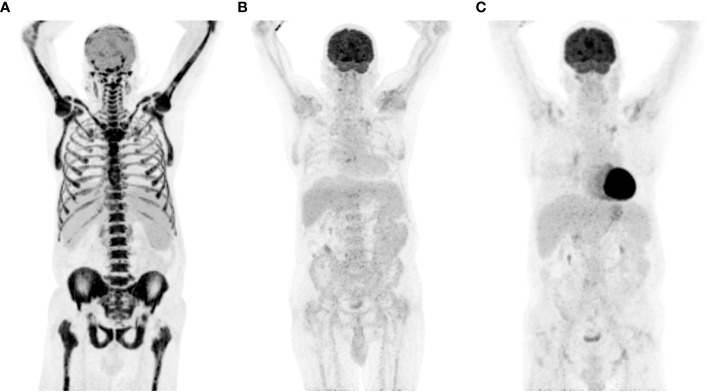
PET-CT response assessment of transformed FL on PD-1 ICB. **(A)** Intense bone marrow diffuse metabolism and mild lymph nodes, at diagnosis of transformation; **(B)** 3-month response assessment; **(C)** 9-month response assessment. FL, follicular lymphoma; ICB, immune checkpoint blockade; PD-1, programmed death-1; PET-CT, positron emission tomography and computed tomography; SUV max, maximum standardized uptake value.

Based on these findings and informed by published results from a small phase 2 study in Richter syndrome (1), the patient received one cycle of rituximab (375 mg/m^2^) and cyclophosphamide (1,000 mg/m^2^), followed by pembrolizumab 200 mg and rituximab, administered every 3 weeks. Astonishingly, the patient’s overall condition improved dramatically, culminating in a complete metabolic response at the 3-month mark (**Figure 3**).

In total, the patient received 24 months of pembrolizumab therapy and maintained a sustained clinical and metabolic remission. Regrettably, despite being in remission, the patient tragically succumbed to septic shock resulting from colitis with paralytic ileus, 3 years after the aggressive transformation from FL.

## Discussion

Despite promising responses to therapies like anti-CD20 immunotherapy and targeted agents, FL often relapses, especially when eligible cell therapies are unavailable (2). Additionally, FL can transform into a more aggressive lymphoma at an annual rate of 1.5%–2% (3). In our patient’s samples, we detected gene variants crucial for histone modification, including CREBBP, EP300, and KMT2D. These genes are commonly mutated in follicular lymphoma (FL) patients, with KMT2D being the most frequently altered (70%–80%), followed by CREBBP (70%) and EP300 (15%) (4, 5). CREBBP alterations reduce its acetyltransferase activity, affecting histone acetylation, including key proteins like TP53 and BCL6. EP300 encodes a histone acetyltransferase regulating gene expression (4). KMT2D mutations often lead to a loss of function and active transcription marks (5). Approximately 30% of FL patients exhibit genomic changes in genes related to the BCR/NF-κB signaling pathway, such as TNFAIP3 (4). TNFRSF14 gene disruptions are also common, modifying the microenvironment and promoting B-cell expansion in FL (6). Recently, Gao et al. notably reported an association between KMT2D, CREBBP, and TNFRSF14 variants and POD24 in follicular lymphoma (FL). However, to our knowledge, no correlation with transformation has been demonstrated (7).

PD-L1 expression serves as a potential prognostic indicator for immune checkpoint inhibitor (ICI) response (8). Tumor cells use PD-L1 to evade the immune system, influenced by interferon-γ from T cells and NK cells. This variable PD-L1 expression across tumors makes it valuable for assessing an inflamed microenvironment. Although PD-L1 is part of many clinical trials and associated with over 80% of FDA immunotherapy approvals, it predicted responses in only 28.9% of cases, with others lacking predictability or not conducting testing (8). Intriguingly, even in cases with absent PD-L1 expression, notable responses to ICI have occurred. PD-L1 expression, seen at varying levels and assessed using diverse techniques, remains essential in solid malignancies.

PD-1/PD-L1 immune checkpoint blockade (ICB) has been scrutinized in the context of follicular lymphoma (FL), with a distinct study by Armand et al. reporting a notably lower overall response rate (ORR) of 4% (9, 10). In exploring combination approaches, the incorporation of PD-1/PD-L1 ICB with anti-CD20 monoclonal antibodies, whether administered as a single agent or in combination with other therapeutic agents, has demonstrated only modest response rates in relapsed/refractory FL (11–17). Overall, the efficacy and safety of PD-1/PD-L1 ICB in indolent lymphomas, particularly FL, exhibit varying outcomes, emphasizing the need for further research and identification of predictive biomarkers. Similarly, single-agent activity of PD-1/PD-L1 immune checkpoint blockade (ICB) has yielded only modest response rates in patients with relapsed/refractory diffuse large B-cell lymphoma (DLBCL) (18–20). Moreover, PD-1/PD-L1 ICB does not appear to confer substantial benefits when combined with frontline immunochemotherapy in newly diagnosed DLBCL (13, 14, 21) or grade 3b follicular lymphoma (FL) (22). For a comprehensive overview of the efficacy of PD-1/PD-L1 ICB in lymphomas, we recommend referring to our recent review in the field (10).

The correlation between tumor mutational burden (TMB) and immunotherapy response exists but lacks an ideal threshold (23). FDA approval requires a minimum of 10 mutations per megabase (mut/Mb) for pembrolizumab, while FoundationOne designates TMB over 20 mut/Mb as high (24). TMB links to neoantigen abundance recognized through MHC presentation by the immune system (24, 25). However, a TMB exceeding 20 mut/Mb results in only a 45% response rate (6). Factors include immunogenic neoantigen generation, immunoselection favoring antigenic mutant proteins, and the tumor microenvironment affecting T-cell infiltration and activation. TMB’s relation to PD-1/PD-L1 ICB response in hematological malignancies is evolving (10, 26). The median TMB in hematological malignancies is approximately 1.7 mut/Mb, with lymphoma TMB varying due to multiple factors (10, 26). In FL, Chalmers et al. reported a median TMB of 8.3 mut/Mb, with a maximum of 26.7. However, the confidence interval (CI) was notably wide (1.5–9.2), likely attributed to the relatively limited specimen size (n=107). Only 3% of these FL cases exhibited a TMB>20 (24). The same authors also noted a median TMB of 10 mut/Mb among 348 DLBCL samples (with a maximum of 251 mut/Mb), and 18% of cases had a TMB > 20 (24). The conventional TMB cutoffs have mainly been derived from comprehensive cohorts of solid tumors. However, we posit that these benchmarks may not precisely reflect the most appropriate thresholds for hematological diseases. We advocate for a customized, individualized approach for determining TMB thresholds tailored to each specific tumor type. This is particularly pertinent in our case, where we observe a moderate TMB of 6.75 mut/Mb.

Georgiou et al. emphasized that abnormalities within the PD-L1/PD-L2 locus, such as the fusion of PD-L1 with IGH, are detected in approximatively 20% of DLBCLs, with a notable prevalence in the non-germinal center B-cell subtype. They observed that specimens with cytogenetic modifications in the PD-L1/PD-L2 locus, especially those involving translocations or amplifications, exhibited a higher likelihood of manifesting overexpression of PD-L1 at both the mRNA and protein levels (27). However, in our case of DLBCL transformation from a preceding follicular lymphoma (FL), no copy number variations (CNVs) or translocations were detected, providing no apparent explanation for the remarkably positive response to PD-1 immunotherapy.

PD-1 serves as a surrogate marker for T-cell dysfunction, and the restoration of CD8+ T-cell function constitutes the antitumor mechanism of checkpoint blockers. In their study, Zhang et al. successfully elucidated additional mechanisms leading to PD-L1 upregulation in diffuse large B-cell lymphoma (DLBCL) (28). Their findings suggest that targeting the PD-1/PD-L1 immunosuppressive pathway in conjunction with CD73/A2aR inhibitors may offer added clinical benefits and partially overcome primary and secondary resistance to PD-1/PD-L1 blockade (28). In our specific case, the patient received cyclophosphamide shortly before pembrolizumab administration. It could be hypothesized that this distinctive infusion might have induced regulatory T-cell (Treg) depletion, potentially leading to a significant enhancement in antitumor immunity.

This case highlights the complex journey faced by individuals with transformed FL and the potential of immunotherapeutic approaches, even in challenging circumstances. It offers hope for the future of lymphoma treatment. The ongoing quest for reliable markers of response to PD1/PD-L1 immune checkpoint blockade remains a substantial challenge in hematological malignancy patients.

## Data availability statement

The raw data supporting the conclusions of this article will be made available by the authors, without undue reservation.

## Ethics statement

The studies involving humans were approved by the Cantonal Commission for Ethics in Research on Human Beings (CCER), Rue Adrien-Lachenal 8 1207 Genève Tél: +41 22 546 51 01 E-mail: ccer@etat.ge.ch. The studies were conducted in accordance with the local legislation and institutional requirements. The ethics committee/institutional review board waived the requirement of written informed consent for participation from the participants or the participants’ legal guardians/next of kin because the patient was deceased, and previously signed the general institutional informed consent for reuse of his clinical data and material. Written informed consent was not obtained from the individual(s) for the publication of any potentially identifiable images or data included in this article because the patient was deceased, and previously signed the general institutional informed consent for reuse of his clinical data and material.

## Author contributions

NL: Supervision, Writing – original draft, Writing – review & editing. AB: Writing – original draft, Writing – review & editing. SP: Writing – original draft, Writing – review & editing.

## Supplementary material

Supplementary Table 1 PD-L1 scoring formulas. *TPS tumor proportional score, IC tumor infiltrating immune cells, CPS combined positivity score.*

Supplementary Figure 1 Gene panel.
