# Supplementary material for: Case report: From sequence to solution: tailoring treatment for transformed follicular lymphoma (DLBCL) through next generation sequencing study
Source: Front Oncol. 2024 Feb 29;14:1308492. doi: 10.3389/fonc.2024.1308492 (PMC10937737; doi:10.3389/fonc.2024.1308492)
Supplement: Supplementary Table 1 — PD-L1 scoring formulas. TPS tumor proportional score, IC tumor infiltrating immune cells, CPS combined positivity score. [file Image_1.pdf]

ABL1
ABL2
ACVR1
ACVR1B
ACVR2A
ADGRB3
AKT1
AKT2
AKT3
ALK
AMER1
APC
AR
ARAF
ARID1A
ARID1B
ARID2
ARID5B
ASNS
ASXL1
ATF1
ATP1A1
ATR
ATRX
AURKA
AURKC
AXIN1
AXIN2
BAP1
BCL10
BCL11A
BCL11B
BCL2
BCL2L1
BCL2L11
BCL6
BCOR
BCORL1
BCR
BIRC3
BIRC7
BLM
BMPR1A
BRAF
BTK
BUB1B
CALR
CARD11
CASP8
CBFB
CBL
CBLB
CBLC
CCND1

CCND2
CCNE1
CD79A
CD79B
CDC73
CDH5
CDK12
CDK4
CDK6
CDKN1A
CDKN1B
CDKN2B
CDKN2C
CEBPA
CHD4
CHEK1
CHIC2
CIC
CNOT3
COL1A1
COL2A1
CRBN
CREBBP
CRLF2
CSF1R
CSF3R
CSMD3
CTCF
CTNNA1
CTNNB1
CUX1
CXCR4
CYLD
DAXX
DCC
DDB2
DDR2
DDX3X
DEK
DICER1
DNM2
DNMT3A
DROSHA
E2F3
ECT2L
EGFR
EIF4A2
ELF3
EP300
EP400
EPHA3
EPHA7
EPHB1
EPHB6

ERBB2
ERBB3
ERBB4
ERCC2
ERCC3
ERCC4
ERCC5
ERG
ESR1
ETS1
EXT1
EXT2
EZH2
FAM175A/ABRAXAS1
FAM46C/TENT5C
FANCA
FANCC
FANCD2
FANCE
FANCF
FANCG
FAS
FAT1
FBXO11
FBXW7
FGF3
FGFR1
FGFR2
FGFR3
FGFR4
FH
FLCN
FLT1
FLT3
FLT4
FOXA1
FOXL2
FOXO1
FOXO3
FOXP1
FUBP1
FYN
FZR1
GATA1
GATA2
GATA3
GNA11
GNA13
GNAQ
GNAS
GPC3
GREM1
GRIN2A
GRM8

H3F3A/H3–3A
H3F3B/H3–3B
HGF
HIF1A
H3C2/HIST1H3B
HLF
HNF1A
HRAS
HSP90AA1
HSP90AB1
HSPH1
IDH1
IDH2
IGF1R
IGF2
IGF2R
IKBKB
IKZF1
IKZF2
IL7R
ING4
INPP4B
IRF2
IRF4
IRS2
ITGA9
JAK1
JAK2
JAK3
JUN
KCNJ5
KDM5C
KDM6A
KDR
KEAP1
KIT
KLF4
KLF6
KMT2A
KMT2C
KMT2D
KNSTRN
KRAS
LATS1
LATS2
LCK
LRP1B
LTK
MAP2K1
MAP2K2
MAP2K4
MAP3K1
MAPK1
MAPK8

MARK1
MARK4
MCL1
MDM2
MDM4
MED12
MEN1
MET
MITF
MLH3
MMP2
MPL
MRE11A/MRE11
MSH3
MTOR
MUTYH
MYB
MYBBP1A
MYC
MYCL
MYCN
MYD88
MYH11
MYH9
MYOD1
NCOA3
NCOR1
NEDD9
NF1
NF2
NFE2L2
NFKBIA
NFKBIE
NKX2-1
NLRP1
NOTCH1
NOTCH2
NOTCH3
NPM1
NRAS
NSD1
NT5C2
NTHL1
NTRK1
NTRK3
NUP93
NUP98
PAK3
PARK2/PRKN
PARP1
PAX5
PAX8
PBRM1
PDGFRA

PDGFRB
PER1
PHF6
PIK3C2B
PIK3CA
PIK3CB
PIK3CG
PIK3R1
PIM1
PLAG1
PLCG1
PML
PMS1
POLD1
POLE
POT1
PPM1D
PPP2R1A
PPP6C
PRDM1
PREX2
PRKAR1A
PRKCB
PSIP1
PTCH1
PTPN11
PTPN14
PTPRB
PTPRD
QKI
RAC1
RAD21
RAD50
RAF1
RARA
RASA1
RB1
RBM10
REL
RET
RHEB
RHOA
RICTOR
RIT1
RNF213
RNF43
ROS1
RPL10
RPL22
RPL5
RSF1
RUNX1
RUNX1T1
SBDS

SDHA
SDHB
SDHC
SDHD
SETBP1
SETD2
SETDB1
SF3B1
SGK1
SH2B3
SLIT2
SMAD2
SMAD3
SMAD4
SMARCB1
SMARCE1
SMC1A
SMC3
SMO
SNCAIP
SOCS1
SOS1
SOX11
SOX2
SOX9
SPEN
SPOP
SRC
SRSF2
STAG2
STAT3
STAT5B
SUFU
SUZ12
SYK
TAF1
TAF1L
TBL1XR1
TBX3
TCF12
TCF3
TCF7L1
TCF7L2
TERT
TET1
TET2
TGFBR2
TIMP3
TNFAIP3
TNFRSF14
TNK2
TOP1
TRAF7
TRRAP

TSC1
TSC2
TSHR
TTF1
TYK2
TYMS
U2AF1
UBR5
USP8
USP9X
VAV1
VHL
WHSC1/NSD2
WRN
WT1
XPA
XPC
XPO1
XRCC2
ZNF217
ZRSR2
ATM
BARD1
BRCA1
BRCA2
BRIP1
CDH1
CDKN2A
CHEK2
EPCAM
MLH1
MSH2
MSH6
NBN
PALB2
PMS2
PTEN
RAD51C
RAD51D
SMARCA4
STK11
TP53
