# Supplementary material for: Case report: From sequence to solution: tailoring treatment for transformed follicular lymphoma (DLBCL) through next generation sequencing study
Source: Front Oncol. 2024 Feb 29;14:1308492. doi: 10.3389/fonc.2024.1308492 (PMC10937737; doi:10.3389/fonc.2024.1308492)
Supplement: Supplementary Figure 1 — Gene panel. [file Table_1.pdf]

Supplement Table 1. PD-L1 scoring formulas

**PD-L1 scoring formulas**		
**score name**	Mass unit	formula
**TPS**	%	Ratio of the number of PD-L1-stained tumor cells to the total number of viable tumor cells multiplied by 100
**IC**	%	Ratio of the area of tumor infiltrated by PD-L1-stained immune cells to the total tumor area multiplied by 100
**CPS**		Ratio pf the number of PD-L1-stained cells (tumor cells, lymphocytes, and macrophages) to the total number of viable tumor cells multiplied by 100
